# Computing the Structural Dynamics of RVFV L Protein Domain in Aqueous Glycerol Solutions

**DOI:** 10.3390/biom11101427

**Published:** 2021-09-29

**Authors:** Gideon K. Gogovi, Swabir Silayi, Amarda Shehu

**Affiliations:** 1 Department of Mathematics and Statistics, University of Houston—Downtown, Houston, TX 77054, USA; 2 Office of Research Computing, George Mason University, Fairfax, VA 22030, USA; ssilayi@gmu.edu; 3 Department of Computer Science, George Mason University, Fairfax, VA 22030, USA; ashehu@gmu.edu; 4 Department of Bioengineering, George Mason University, Fairfax, VA 22030, USA; 5 School of Systems Biology, George Mason University, Fairfax, VA 22030, USA; 6 Center for Advancing Human-Machine Partnerships, George Mason University, Fairfax, VA 22030, USA

**Keywords:** structural dynamics, molecular dynamics, aqueous glycerol, RVFV

## Abstract

Many biological and biotechnological processes are controlled by protein–protein and protein–solvent interactions. In order to understand, predict, and optimize such processes, it is important to understand how solvents affect protein structure during protein–solvent interactions. In this study, all-atom molecular dynamics are used to investigate the structural dynamics and energetic properties of a C-terminal domain of the Rift Valley Fever Virus L protein solvated in glycerol and aqueous glycerol solutions in different concentrations by molecular weight. The Generalized Amber Force Field is modified by including restrained electrostatic potential atomic charges for the glycerol molecules. The peptide is considered in detail by monitoring properties like the root-mean-squared deviation, root-mean-squared fluctuation, radius of gyration, hydrodynamic radius, end-to-end distance, solvent-accessible surface area, intra-potential energy, and solvent–peptide interaction energies for hundreds of nanoseconds. Secondary structure analysis is also performed to examine the extent of conformational drift for the individual helices and sheets. We predict that the peptide helices and sheets are maintained only when the modeling strategy considers the solvent with lower glycerol concentration. We also find that the solvent-peptide becomes more cohesive with decreasing glycerol concentrations. The density and radial distribution function of glycerol solvent calculated when modeled with the modified atomic charges show a very good agreement with experimental results and other simulations at 298.15K.

## 1. Introduction

The Rift Valley Fever Virus (RVFV) was first discovered in 1931 in the Great Rift Valley of Kenya, East Africa [1]. It is anarbovirus in the Bunyavirales order, Phenuiviridae family and Phlebovirus genus. Even though it was discovered in East Africa, it has spread and caused periodic outbreaks in human and livestock populations throughout Africa, and as far as into the Arabian Peninsula. The virus is vectored by mosquitoes and, as such, outbreaks tend to follow periods of heavy rainfall that significantly increase mosquito populations [1]. RVFV infects ruminants and pseudo-ruminants, leading to abortions in pregnant animals and high mortality rates among young animals [2]. Because of the high economical burden of RVFV outbreaks, the possibility of severe disease without effective antiviral treatment options and the epidemic potential, the World Health Organization (WHO) has urged that Research and Development (R&D) efforts focus on this pathogen to develop medical countermeasures [3].

Since RVFV is a negative-sense RNA virus that contains three segments of viral RNA—the S, M, and L segments, it can also be transmitted to humans and cause febrile illness with the possibility of severe disease [4]. Structurally, the full RVFV L protein is made up of a sequence of 2092 amino acids and is also known to have flexible termini of about 200 amino acids each, with a high proportion of helical regions [5]. The structure of the C-terminal of the RVFV L protein, a 117 amino acid-long domain, has been recently modeled using X-ray crystallography. The domain has been shown to be highly similar to the PB2 cap-binding domain of the influenza virus and to the putative non-functional cap-binding domain of reptarenaviruses [6].

We know that the dynamics and functions of proteins are coupled to the motion of solvent molecules [7,8,9]. There is a high interest in the investigation of protein-solvent interactions, because many biological and biotechnological processes are controlled by protein–solvent and/or protein–protein interactions. In order to better understand, predict, and optimize such processes, it is valuable to understand how solvents of different concentrations affect protein-solvent interactions. Computational studies of the protein–solvent interactions have generally been performed using the Langevin mode analysis [10], molecular dynamics simulations(MD), and normal mode analyses (NMA) [8,9,11,12].

NMA provides a complete analytical solution of the vibrational modes of a system at its conformation of local energy minima [13] and decomposes all the possible motions of protein and solvent atoms together with their corresponding frequencies. This is the method often used to analyze the protein–water interactions from MD trajectories [8,9]. This is because, in the course of the MD simulations, it is possible to observe the time evolution of the interfacial dynamics of complex molecular structures, either localized around particular macromolecules or interacting with molecular liquids. Such details are not yet observable experimentally [14]. They can, however, be determined computationally through an all-atom modeling of the components using MD for analysis of the system thermodynamics, the proteins’ structural dynamics, and the energetic properties. MD simulations of proteins with explicit solvent molecules provide abundant and detailed atomic motions and interactions.

A number of experimental techniques, including densimetry, neutron scattering, and dielectric relaxation [15,16,17], have been employed for a range of cosolvents in order to determine whether proteins are preferentially solvated by a specific solvent or by its cosolvent. It has been shown that the structural stability and biochemical activity of proteins can be dramatically affected by the addition of cosolvents to aqueous protein solutions [18,19]. The relative abundance of each solvent in the solvation shell of proteins in solvent mixtures has also been shown to have a critical impact on their properties [20]. Computationally, local solvation preferences of a protein can be quantified over the entire surface from extended MD simulations [21]. Even though some cosolvents denature proteins, others preserve protein structure. Solvents such as urea are denaturants, and polyols like glycerol and sugars are protectants [22,23].

Glycerol has a complex conformational space because of its high flexibility and the presence of vicinal hydroxyl groups that are capable of stabilization through intramolecular hydrogen bonds. Glycerol may also exist as a super cooled liquid, a property that makes its crystallization possible only through special techniques [24]. In vitro glycerol helps preserve biomolecular structure and also enhances the self-assembly of biomolecules [24]. A number of studies have demonstrated how glycerol promotes protein folding and prevents aggregation. However, we still lack a detailed understanding of the mechanism through which glycerol improves or affects protein stability. For example, Ou W. et al. [25] studied the effects of glycerol in the refolding, reactivation, unfolding, and inactivation of guanidine-denatured creatine kinase by observing the fluorescence emission spectra, the circular dichroism spectra, and by the recovery and inactivation of enzymatic activity and aggregation. Results from the study showed that low concentrations of glycerol (<25%) improve the refolding yields of creatine kinase, but high glycerol concentrations decrease its recovery. Glycerol also favors the secondary structural formation and inhibits the aggregation of creatine kinase, as proline does. In another study by Rariy and Klibanov [26], unfolded and reduced hen egg-white lysozyme was refolded and reoxidized in glycerol containing varying amounts of water. A densimetric investigation of the interactions between the solvent components in the glycerol-water mixtures (between 10–40 by vol% glycerol) and seven proteins carried out in the acid pH region showed that all the proteins were consistently preferentially hydrated in all cases. This was expected, since such thermodynamically unfavorable interactions (addition of proteins to the mixed solvent) result in an increase in the glycerol chemical potential. Such interactions also tend to minimize the surface of contact between proteins and glycerol, and in this way, stabilize the native structure of globular proteins [15]. M. Farnum and C. Zukoski [27], in a related study, investigated glycerol and ionic strength effects on the solubility and strength of interactions of bovine pancreatic trypsin inhibitor. The two variables in their study were found to have opposite effects on the intermolecular forces. Attractions increased with NaCl, whereas repulsions increased with glycerol concentration. The bovine pancreatic trypsin inhibitor follows the same general phase behavior as other globular macromolecules. In these, a robust correlation between the protein solution second virial coefficient and solubility has been developed. MD and comparative structural analyses of magainin in pure water, glycerol/water, 2,2,2-trifluoroethanol/water, and sorbitol/water [28] indicate that glycerol and sorbitol molecules decrease the interactions of water molecules with the hydrophobic residues of the peptide, while at the same time, stabilize the alpha helical structure.

Even with multiple perspectives and results from several studies including wet-laboratory experiments on different proteins, protein structural dynamics in solvents still remain comparatively less well understood. From a biological perspective, domains of the RVFV L protein have very similar characteristics to other proteins. It is therefore important to have a visual understanding of the structural dynamics of the RVFV L protein domain in explicit solvents of different densities and concentrations. This requires a molecular-level understanding of the protein dynamics in these varied environments, since a comprehensive molecular picture of protein (de)stabilization by co-solvents has so far remained elusive. There are currently no FDA-approved vaccines or therapeutics to prevent or treat RVFV infection in humans or ruminants [29]. Therefore, the findings presented here on the structural dynamics of the L protein domain could facilitate studies of other protein–protein and protein–solvent interactions and may also represent new targets for therapeutic design. Understanding the conformational ensemble of the cap-binding state without a binding partner could reveal novel targets not observed in static structures of the cap-binding domain. This will, in turn, aid in the design of therapeutics targeting this important binding domain.

In this study, we focus on a C-terminal domain of the RVFV L protein. We conduct extensive all-atom MD simulations of the RVFV L protein domain in glycerol and aqueous glycerol solutions in an attempt to better understand the behavior of the domain in glycerol solution. We look at the structural dynamic changes of the domain in the solvents at different concentrations by molecular weight. This study is significant, because glycerol is known to shift the native protein ensembles to more compact states. It also inhibits aggregation during refolding [30].

The rest of this paper is organized as follows. Following this section is Section 2, where the computational approaches and methods employed in this study are described in detail. In Section 3, we elaborate on the results obtained from the MD simulations. We present the conclusions from the study in Section 4.

## 2. Materials and Methods

The methodology explores two scenarios. Before solvating the protein domain in glycerol and its aqueous solutions, we validate simulations of the pure glycerol solvent and the aqueous mixtures. In the first scenario, the focus is on modeling the explicit solvents with restrained electrostatic potential atomic partial (RESP) charges under ambient conditions. The second scenario focuses on the solvation of the protein domain to study the effect of these solvents modeled within the all-atom explicit solvent on the protein domain.

### 2.1. Methodology for the All-Atom MD Simulation of Aqueous Glycerol Solutions

Ten different solvents of pure glycerol and glycerol:water at 90:10, 80:20, 70:30, 60:40, 50:50, 40:60, 30:70, 20:80, and 10:90 percentage concentrations in molecular weights are considered in this study. The solute in each of these solvents is the C-terminal domain from the RVFV L protein. The simulation box sizes are different for each system size because of the different number of atoms. This is also in accordance with the respective system densities. The general AMBER force field (GAFF) [31,32] was used to generate atomic charges for a glycerol molecule parameterized to reproduce the B3LYP 6-31G* charges. This includes the polarizable continuum model (PCM) [33], which is based on the Merz–Singh–Kollman population analysis [34,35], and is done using Gaussian09 [36]. The atomic charges are then ported into the AMBER Tools18 [31] to generate the corresponding RESP values, which are employed in this study for the glycerol component.

Glycerol is a liquid between 291 K and 563 K, and it is often used mixed with water in a large variety of relative concentrations. The simulations of pure glycerol contain 3000 molecules, while for the 90:10 glycerol:water mixed systems, the simulations involve 2700-1527 glycerol–water molecules, and the 80:20, 70:30, 60:40, 50:50, 40:60, 30:70, 20:80, 10:90 have 2400-3054, 2100-4581, 1800-6108, 1500-7635, 1200-9162, 900-10689, 600-12216, 300-13743 glycerol–water molecules, respectively. These systems are equilibrated with NPT-MD at 298.15 K and 1.01325 via the Langevin thermostat with a collision frequency of 5 ps^−1^ and a time step of 1 fs along a minimum of 20 ns long trajectories using a 14 Å cutoff distance with periodic boundary conditions (PBC). The volumes of the systems that gave the respective equilibrium densities are presented in the Supplementary Material as periodic box size per side. Ewald sums are used in all calculations for the long-range electrostatics within the particle mesh implementation (PME). Prior to the NPT-MD simulations, all the solvent boxes are thermalized with NVT-MD simulations for 10 ns after relaxing the systems with energy minimization. We run a total of 60,000 steps of minimization; 50,000 of steepest descent method followed by 10,000 of the conjugate gradient to relax the systems. The SPC/E water model [37], which is known from a previous study [38] to preserve the structure of the RVFV L protein domain, is used for the water component. Once we achieve the equilibrium density using the NVT-MD simulation, we follow up with an NVE-MD production run for 10 ns at temperatures around 298 K for the same system. In this way, we are able to calculate the diffusion coefficients of 100% glycerol. To calculate the diffusion coefficients, the centers of mass of the glycerol molecules are tracked in time within the solution and the diffusion coefficient values determined from Equation (1).

(1) $$D=\frac{1}{6t}\frac{1}{m}\sum_{k=1}^{m}\frac{1}{N}\sum_{i=1}^{N}{\left(r_{i}\left(t\right)-r_{i}\left({t_{0}}_{k}\right)\right)}^{2}+D_{PBC}$$

where $r_{i}$ is the position of the ith molecule center of mass at time *t* and *N* is the number of molecules in the solvent. Each NVE run is split into *m* time series. Each of the runs starts from a reference position $r_{i}\left({t_{0}}_{k}\right)$, and their average is taken as indicated in Equation (1). The last term is the correction due to the periodic boundary conditions [39], $D_{PBC}=\frac{2.837297k_{B}T}{6\pi \eta L}$, with $k_{B}$ being Boltzmann’s constant, *T* temperature, *L* computational box length, and η solvent viscosity. The value for the η is taken from an experiment at 298 K: $\eta_{glycerol}$ = 945 mPas [40].

### 2.2. Methodology Associated with All-Atom MD Simulation of RVFV L Protein Domain in Solvent

The next step is the preparation of systems with the protein domain solvated into each of the ten solvents. The starting coordinates of the peptide were taken from the X-ray crystallographic structure (PDB ID: 6QHG) [6]. This domain of the L protein is composed of 117 amino-acids (G1706—K1822). In order to avoid an artificially strong interaction between termini, which is not natural considering the “real life” environment of the sequence, we add ACE/NME capping to the protein domain. The N-terminal residue is taken to be a capping acetyl group (ACE) and the C-terminal, N-methyl amide capping group (NME). This is achieved using the tleap program in AMBER during the topology and parameter file creation stage. The systems are then relaxed with a 60,000-step steepest descent method energy minimization, followed by 15,000 steps of the conjugate gradient method. MD simulations of the domain in the different solvent concentrations were carried out using the AMBER 18 package with the ff14SB force field [41]. The systems were equilibrated with NVT-MD at the volume that gave the respective equilibrium densities for hundreds of nanoseconds at 298.15 K via the Langevin thermostat with a collision frequency of 5 ps^−1^ and a time step of 1 fs using a 16 Å cutoff distance. This was followed by several nanoseconds of production runs. It is from the last 200 ns of these NVT-MD simulations that the energetic, structural, and dynamic properties of the solvated domain are then calculated. We also use the Kabsch and Sander procedure [42] of protein secondary structure analysis to study the secondary structure elements of the domain in the solvents. Post-simulation processing and analysis of the MD trajectories data were performed with CPPTRAJ [43] and, in some cases, with in-house written Fortran and bash scripts.

## 3. Results and Discussion

### 3.1. Properties of All-Atom MD Simulated Solvents

**Figure 1:**
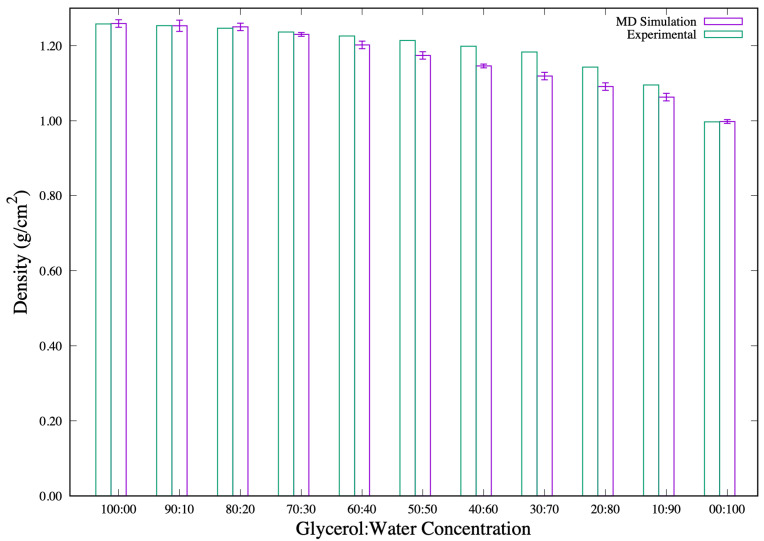
Densities, ρ (g/cm${}^{3}$) of glycerol and glycerol-water ($x_{1}$:$x_{2}$) mixtures at 298.15 K and atmospheric pressure.

The glycerol solution simulations are performed in order to quantify the appropriate behavior of the solvents at 298.15 K. From the NPT-MD simulations, the solvent systems attain equilibrium densities as presented in Figure 1. A table of these density values is presented in the Supplementary Material. These densities, presented as a function of glycerol concentrations, are in good agreement with experimental glycerol solution densities [44] at 298.15 K. This comes out clearly in the 100% to 60% glycerol concentration systems. It is worth mentioning that our calculated densities from the solvents composed of 50% to 10% glycerol produced higher deviations from the experimentally measured densities. However, the calculated densities presented here are much closer to the experiment than those reported in other simulation studies’ [45] for similar concentration proportions. The density of the 100% SPC/E water model adopted from [38] is also shown in Figure 1 as 00:100 for easy comparison.

**Figure 2:**
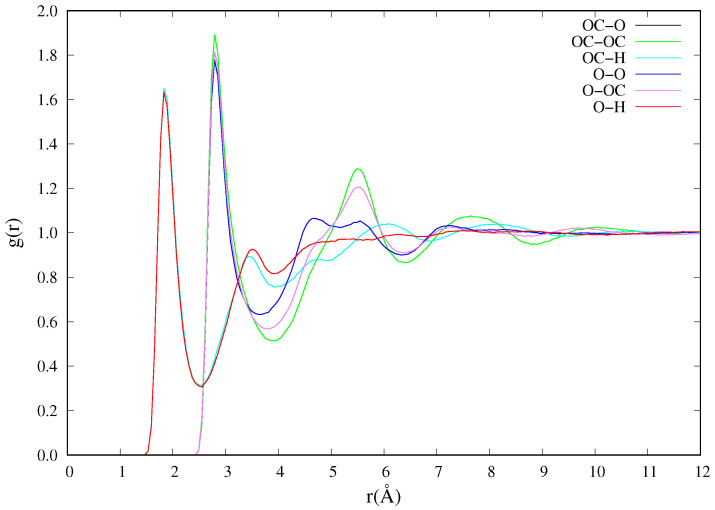
Radial distribution function of glycerol at 298 K and equilibrium density 1.259 g/cm${}^{3}$/.

The radial distribution function, rdf or g(r), of the glycerol solvent is calculated and compared with experimental observations at the same temperature and equilibrium densities. This too shows very good agreement with experimental results. The calculated peak positions for the six atom pairs (O−H, OC−O, OC−OC, OC−H, O−O, O−OC) depicted are 1.85, 1.84, 2.80, 2.80, 2.80, 2.80 Å, which are in excellent agreement within standard deviation with experimental values of 1.77±0.61, 1.80±0.63, 2.73±0.87, 2.76±0.78, 2.76±0.80, 2.76±0.90 Å [46]. Figure 2 shows the rdf of glycerol between atom pairs in each glycerol molecule. Another solvent property of interest is the self-diffusion coefficient. The self-diffusion coefficient of the pure glycerol is calculated from Equation (1) by considering 40 different time origins, each of which is a 0.5 ns NVE-MD time evolution simulation. The PBC corrected self-diffusion coefficient of glycerol calculated from the simulation at 298±1 K is ($1.93\pm 0.02)\times 10^{-7}$ cm${}^{2}$/s. This calculated diffusion coefficient compares well with the experimental value of $1.7\times 10^{-7}$ cm${}^{2}$/s obtained from the Taylor dispersion method [47] and another simulation with a value in the order ($\times 10^{-7}$) using the AMBER force fields [48] at approximately 298.51 K.

This experimental value is larger than the diffusion coefficient obtained from the NMR pulsed magnetic field gradient [49] or the modulated gradient spin echo method [50]. We therefore have a reason to assume that our force field is modeling the glycerol solutions adequately at 298.15 K for the goals of this work.

### 3.2. Energetic Evaluation of RVFV L Protein Domain

In Table 1, we present an energetics evaluation of the protein from the various solvent concentrations. The average potential energy of over the last 200 ns of the simulations shows a decreasing potential energy as glycerol concentration decreases. This indicates an increasing stabilization of the protein domain with decreasing glycerol concentration. The interaction energy, also shown in Table 1, between the peptide and the solvents represents the balance between total potential energy of the system and the sum of individually separated potential energies of the solvent and the peptide: $E_{\mathrm{int}}=E_{\mathrm{full}-\mathrm{system}}-\left(E_{\mathrm{solvent}}+E_{\mathrm{peptide}}\right)$. As observed in the table, the solvent–peptide interaction energy in each of the solvents becomes more cohesive as the concentration of glycerol decreases. This indicates that the water-dominant solvents stabilize the structure, leading to a smaller radius of gyration distribution.

From this, it can be deduced that the interaction energies of the protein in aqueous glycerol solution depend on the glycerol concentrations. The peptide stabilization propensity increases as the number of water molecules increase in the solvent. The large fluctuations in the energies of systems with higher glycerol concentrations can be attributed to the mobility of the few water molecules in the neighborhood of the peptide. This mobility results in frequent changes in the solution surrounding the solute, and register as larger fluctuations.

### 3.3. Properties of RVFV L Protein Domain in the Solvents

**Figure 3:**
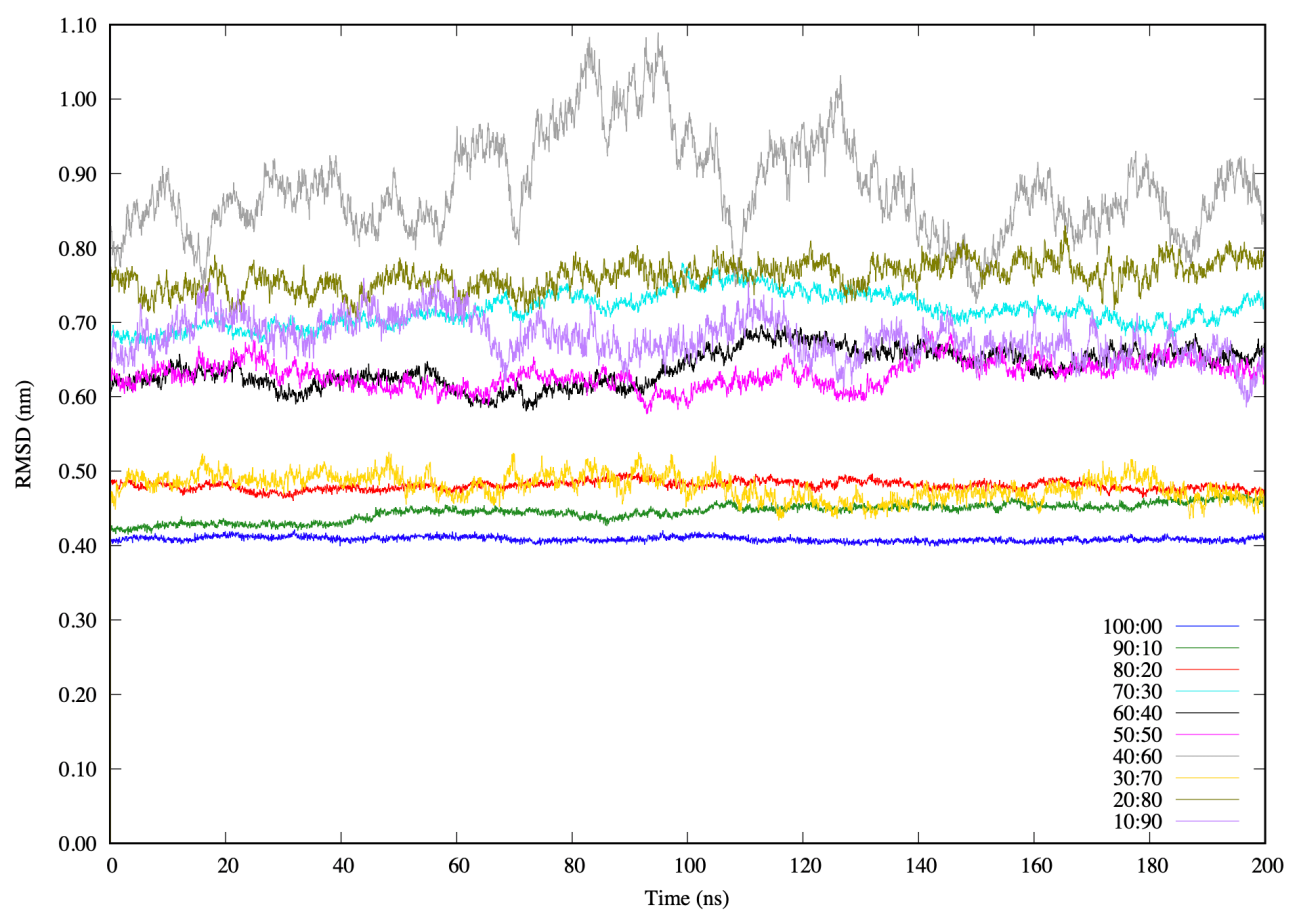
Conformational change in the RVFV protein domain measured as the root-mean-squared deviation (*RMSD*).

In addition to the energetics evaluations, several structural properties of the domain and the solvents were monitored during the simulations. An analysis of the various MD trajectories indicates that the structural impressions of the solvated protein are acquired in the course of the simulations. The calculated structural properties of the protein domain include root-mean-squared deviation (RMSD), root-mean-squared fluctuation (RMSF), radii of gyration $\left(R_{g}\right)$ and hydrodynamic $\left(R_{hyd}\right)$, end-to-end distance $\left(R_{e-e}\right)$, and the solvent-accessible surface area (SASA). Some of the average values of these calculated structural properties, presented in the Supplementary Material, are very similar (within a specific property for the domain) across the different solvent concentrations. Examining the RMSD as a function of time for all residues in the protein together, it can be seen that there is a relative rise between 0.4 nm–0.9 nm, with some fluctuations along the last 200 ns of each simulation as shown in Figure 3. A large increase occurs in the Cα positional RMSD value in the solvents containing 40% water molecules, indicating how much the entire protein moves away from the starting conformation. This, however, was different for the 30:70 system (yellow), which behaves like the three systems with higher glycerol concentrations, but has additional considerable fluctuations in RMSD. The instability in the water-dominant systems is similar to other proteins in water, as observed in other simulations [51,52]. Similar to another MD simulation study [28], our simulations have shown that the RMSD is increased by adding co-solvents to the glycerol solutions. This is significant, as it is an indication that solvents constrain Cα movement with respect to the initial structure.

In addition to indicating positional differences between the different structures over time from RMSD, we also investigated individual residue flexibility, or how much a particular residue moves (fluctuates) during a simulation.

**Figure 4:**
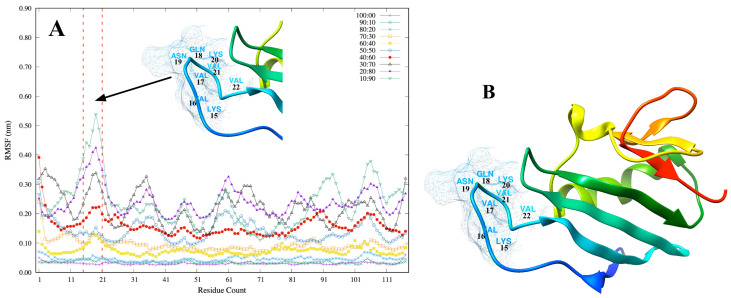
Individual residue flexibility in the RVFV protein domain measured as the RMSF. (**A**) calculated protein RMSF values from all the solvent concentrations; (**B**) a snapshot of the entire domain with a mesh and residue name labels for the highly flexible region.

This is achieved by calculating the RMSF from the equation.

$$RMSF_{i}=\sqrt{\frac{1}{T}\sum_{t_{j}=1}^{T}|r_{i}\left(t_{j}\right)-r_{i}^{ref}{|}^{2}}$$

where *T* is the time over which one wants to average and $r_{i}^{ref}$ is the reference position of particle *i*. This reference position is the time-averaged position of the same particle *i*. The calculated protein RMSF values from all the solvent concentrations are presented in Figure 4A. The relatively small total positional difference observed in the 100:00 and 90:10 systems is also evident from the RMSF calculations. However, in the other systems, a high evidence of individual residue flexibility is observed in the region containing the sequence K15 -V22 (KVVQNKVV). This is indicated in Figure 4A in the section of the RMSF with the two vertical red dashed lines. Figure 4B also shows a snapshot of the entire domain with a mesh and residue name labels for the highly flexible region. This flexible region, K15-V22, of the domain obtained from the RMSF calculation is found within a hydrophobic pocket of two aromatic amino acid side chains, F13 and K28, determined experimentally by [6] as a place where 7-methylguanosine 5′-triphosphate (m${}^{7}$GTP) molecule bounds. This is typical for cellular and viral cap-binding proteins. In developing potentially broad-spectrum inhibitors against the RVFV and other viruses from the *Bunyavirales* order, the region K15-V22 of the cap-binding cavities is important.

We calculate the SASA, which is the surface area of a bio-molecule that is accessible to a solvent, and observe that it is decreased in systems with lower glycerol concentrations. This indicates that, as water molecules increase in count, the peptide surface exposed to the solvent decreases. Under the conditions studied, the domains have SASA sizes between 963.39±6.46 nm${}^{2}$ and 758.29±39.41 nm${}^{2}$. In contrast to natively folded proteins, intrinsically disordered proteins generally lack well-defined 3D structures. Consequently, they explore a large number of distinct conformations, and their conformational properties are thus best described in statistical terms.

One useful and informative way of representing this large conformational ensemble is through a distribution of the $R_{g}$, calculated with the Equation $R_{g}^{2}=\sum_{i=1}^{N}{\left(r_{i}-r_{cm}\right)}^{2}/N$ and the hydrodynamic radius, $R_{hyd}$, calculated from $\frac{1}{R_{hyd}}=\frac{1}{N^{2}}\sum_{i=1}^{N-1}\sum_{j>i}^{N}\frac{1}{r_{ij}}.$ The $R_{hyd}$ is an approximation of the Stokes radius measurable through size-exclusion chromatography.

In both the radii formulae above, $r_{i}$ are atomic position vectors relative to the protein center of mass, $r_{cm}$ is the center of mass position vector, $r_{ij}$ are distances between atoms *i* and *j*, and *N* is the number of atoms in the protein. The ensemble averages give an idea of the degree of the protein compactness and may be compared to the values for other proteins of similar lengths. A common feature observed in the $R_{g}$ across the glycerol concentrations in the course of the simulations is that the protein gives rise to very compact $R_{g}$ distributions when compared between the solvent concentrations. We conclude from this that the RVFV L protein domain prefers to remain compact in glycerol dominant solvents. This is consistent across the different glycerol concentrations and across the simulations at temperature 298.15 K. The solvent molecules trap the instantaneous geometry, and the fate of the protein is locked in an instantaneous structure. When simulated in the water-dominant solvents, the domain exhibits a bit more flexibility. Because both $R_{g}$ and $R_{hyd}$ probe the compactness of disordered proteins, and because they may contain complementary information about the distribution of states [53], there have been several studies on the relationship between $R_{g}$ and $R_{hyd}$ for disordered proteins and polymers [53,54,55,56]. In line with theoretical expectations, it was found that the ratio $R_{g}/R_{hyd}$ depends substantially on the compaction of the protein chain, so that compact states have ratios ≈ 0.77 or ${\left(3/5\right)}^{1/2}$ [54]. When molecules deviate from globular to non-spherical or elongated/extended structures, the observed $R_{g}/R_{hyd}$ tends towards values away from ${\left(3/5\right)}^{1/2}$. Because of the relative level of compactness of the chain, when quantified by $R_{g}$, which also depends on the chain length, the ratio $R_{g}/R_{hyd}$ also depends on the number of residues (N) of the protein. These two effects were combined into a single, physically-motivated and empirically parameterized equation that enables one to calculate $R_{hyd}$ for a configuration of an intrinsically disordered protein from its $R_{g}$ [57] using the relation:

$$\frac{R_{g}}{R_{hyd}}\left(N,R_{g}\right)=\frac{\alpha_{1}\left(R_{g}-\alpha_{2}N^{0.33}\right)}{N^{0.60}-N^{0.33}}+\alpha_{3}$$

where $\alpha_{1}$, $\alpha_{2}$, and $\alpha_{3}$ are parameters that are fitted to maximize agreement between the model and hydrodynamic calculations. The $R_{g}/R_{hyd}$ values calculated from the simulations in this study for the different solvent concentrations show that the RVFV L protein domain maintained its non-spherical shape, with a $R_{g}/R_{hyd}$ ratio in the range $0.498\leq R_{g}/R_{hyd}\leq 0.532$. Another useful property is the end-to-end distance, $R_{e-e}$, defined as the distance between the two end residues of the protein chain. The $R_{e-e}$ describes the flexibility of the protein domain.

### 3.4. Secondary Structure Analysis of the RVFV L Protein Domain

Having established that the degree of conformational change in the protein domain is modest in the solvent environment, and having shown that it is comparable to that in simulations of other proteins [58], it is informative to examine the extent of conformational drift for the individual helices and sheets. To investigate this, secondary structure analysis was carried out on the domain in the solvents. The α-helices, β-sheets, and turns are the common secondary structures in proteins with the common element of most of these structures being the presence of characteristic hydrogen bonds. Because their backbone ϕ and ψ angles repeat, helices are classified as repetitive secondary structure. Alternatively, if the backbone dihedral angle pairs are the same for each residue, the resulting conformation will assume a helical conformation about some axis in space [59]. The β-sheets are another major structural element in globular proteins [42,60] and are found in two forms, parallel or antiparallel, based on the relative directions of two interacting beta strands. The basic unit of a beta sheet is a β strand with approximate backbone dihedral angles ϕ=−120 and ψ=+120, producing a translation of 3.2 to 3.4 Å/residue for residues in antiparallel and parallel strands, respectively. Due to the more optimal orientation of the interstrand hydrogen bonds, antiparallel β-sheets are thought to be intrinsically more stable than parallel sheets. Hydrogen bonds in a parallel β sheet are not perpendicular to the individual strands, resulting in components parallel to the strand [61].

**Figure 5:**
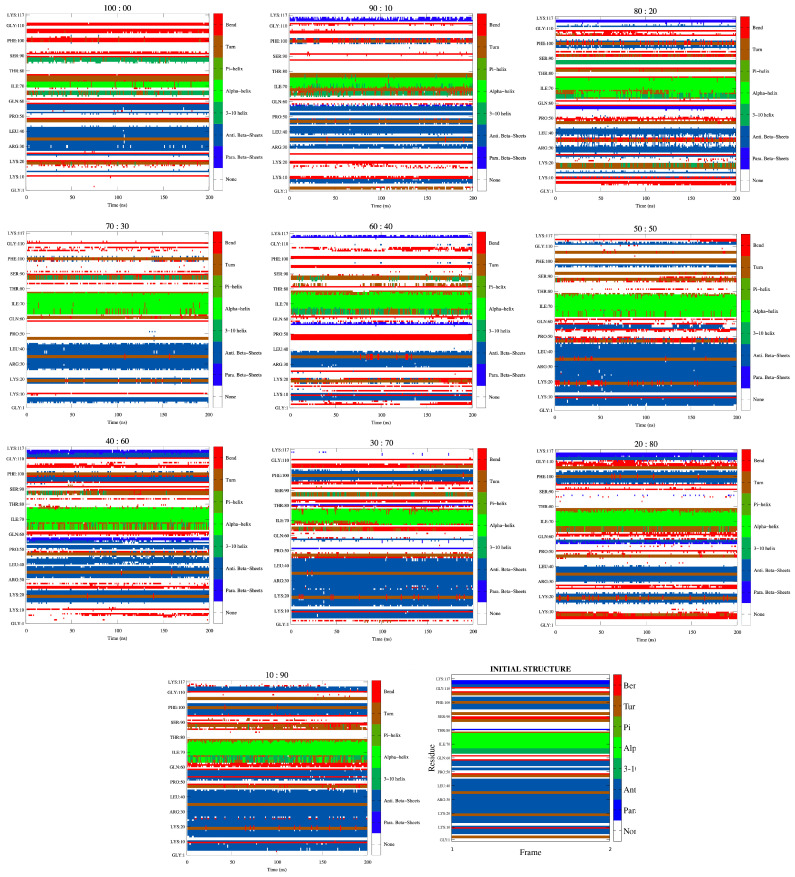
Secondary structure analysis (via the Kabsch and Sander procedure [42]) of the RVFV L protein domain at 298.15 K in the pure glycerol, and aqueous glycerol solutions.

Figure 5 presents results from the secondary structure analysis of the protein domain. We find that, in general, the structure does not change much from the initial configuration with time. Comparison of the 100% glycerol with glycerol/water solutions, however, shows some amount of difference. Compared to pure glycerol, the largely α-helical conformation of the peptide is maintained throughout the last 200 ns of simulations. Some local deviations from α-helicity were observed in the C- and N-termini of the protein. The analysis of secondary structure elements also showed that the helices in this region of the L protein are relatively stable in the different glycerol concentrations. Their stability increases as the glycerol concentration decreases. This stability trend implies that the observed conformational changes or the large RMSD values observed are not generally caused by an unfolding of the structure. It therefore becomes reasonable to conclude instead that in this cap-binding domain of RVFV L protein, relatively larger motions occur as the glycerol concentration decreases, or when the solvent density decreases. It can also be concluded that the observed flexibility is inherent to the structure. Conformational change appears to originate from an opening of the helix-loop regions. The secondary structure analysis also shows some preservation of the β-sheets along the course of the simulation in all solvents with partial disappearance in some residues.

### 3.5. Investigating the Linear Relationship between $R_{hyd}$ and $R_{g}$ with Cluster Analysis

Clustering is a general machine learning technique that can be applied to any collection of data elements or points where a function measuring distance between pairs of points is available [62,63]. This technique is used here to further investigate the linear relationship between the $R_{hyd}$ and $R_{g}$ within structures of similar conformations. The clustering algorithm partitions the data points into a disjointed collection of clusters. The points within one cluster are ideally closer, or more similar, to each other than to points from other clusters. The use of clustering algorithms to group similar conformations observed during an MD simulation is not new [64,65]. In this case, we used the clustering technique to group similar conformational structures visited during the simulation into groups.

A subset of publications developing and applying machine learning algorithms to analyze MD trajectories covers some of the earliest applications of these methods to MD simulations to very recent studies [64,66,67,68,69,70]. When clustering the molecular configurations from an MD trajectory, each clustering algorithm should ideally group similar molecular configurations into distinct sets or groups. This gives a refined view of how a given molecule is sampling conformational space and allows direct characterization of the separate conformational substates visited by the MD simulation [71]. It is worth noting here that large-scale conformational changes during the MD simulation can lead to high variance in the calculation of time-independent properties, such as the estimation of free energetics [72]. By clustering the trajectory into distinct sub-state populations, we can minimize this variance and provide more useful information about the ensemble of conformations sampled by MD.

**Figure 6:**
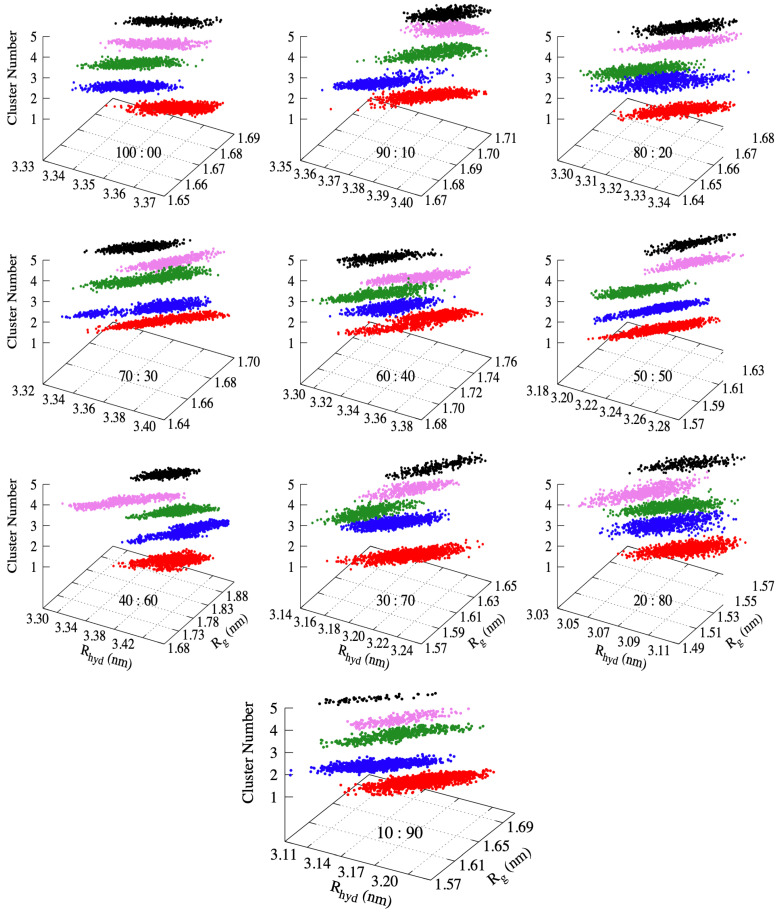
Cluster distribution along the MD trajectory of the RVFV domain from the hierarchical agglomerative clustering algorithm. The radius of gyration vs hydrodynamic radius of peptide over the trajectory which is colored based on their cluster memberships along the 200 ns MD runs at 298.15 K.

This work applied the well-known pairwise distance metric clustering algorithm, Agglomerative Hierarchical Clustering, to the MD trajectories. The bottom-up hierarchical clustering approach is employed to cluster the trajectories of the RVFV L protein domain solvated in the glycerol and its aqueous solutions. The clustering on the backbone atoms of the protein using average-linkage with a stopping, when either 5 clusters are reached or the minimum euclidean distance, d(p,q)=$\sqrt{{\left(p_{x}-q_{x}\right)}^{2}+{\left(p_{y}-q_{y}\right)}^{2}+{\left(p_{z}-q_{z}\right)}^{2}}$, between clusters p and q is 0.3 nm, was used. A visualization of the results obtained from the clustering analysis is presented in Figure 6 as a graph of $R_{hyd}$ versus $R_{g}$. The individual clusters are represented by the different colors across the solvent concentrations. In all the systems, 5 clusters were obtained based on the stopping criteria used in the clustering. More importantly, the linearity relationship between the $R_{hyd}$ and $R_{g}$ is observed within structures with similar conformations (see Figure 6).

A comparison of cluster sizes, average distances of each conformation in the cluster to its centroid, and average distances between the clusters within each solvent is calculated. The average distance of each conformation in the cluster to the centroid of the cluster spans a large range. The range is from 0.103±0.004 nm to 0.393±0.023 nm with the larger distances observed in the water-dominant solvents. This result further explains the large RMSD and $R_{g}$ values obtained earlier in the water-dominant solvents. The result also reflects the relevance of understanding the transport mechanism of the RVFV domain in aqueous glycerol environment.

## 4. Conclusions

In this work, we have presented a computational investigation of the structural dynamics and energetics of the RVFV L protein domain in glycerol solutions with the goal of understanding and explaining the sensitivity of the peptide to such viscous liquids. We find that the solvent concentrations do have some effect on the conformation of the protein domain with conformation change increasing as concentration of water increases. The structural conduct and preference of the domain is found to be less sensitive to the solvent environment containing higher glycerol molecules. These effects play an important role in protein folding in the presence of glycerol. We further demonstrate that the structural dynamics of the domain are maintained when the modeling strategy considers solvents with high glycerol concentration. From this, we can conclude that the protein structures studied here undergo relatively small conformational changes in solutions of high glycerol concentration as compared to water-dominant solutions.

Peak positions of the radial distribution function for the glycerol solution as calculated with the modified generalized amber force field by including the restrained electrostatic potential atomic charges for the glycerol molecules show very good agreement with the experimental results at 298.15 K. The calculated densities, however, only showed a good agreement with experimental values for the higher concentration (100% to 60%) glycerol systems. Solvents composed of 50% to 10% glycerol produced higher deviations from the experimentally measured densities. The structures computed from solvents with higher water concentrations exhibited a strong attraction between the protein and solvent molecules. This indicates that the solvent-proteins become more cohesive with decreasing glycerol concentrations. We predict that the protein domain only maintains the α−helices and β−sheets when the modeling strategy we employed considers solvents with less glycerol concentrations. Finally, the study identifies a flexible region in the domain within a hydrophobic pocket of aromatic amino acid side chains that had recently been discovered experimentally as a bonding place for m${}^{7}$GTP molecule. This region of cap-binding will be important in developing broad-spectrum inhibitors against the RVFV and other viruses from the *Bunyavirales* order.

## Figures and Tables

**Table 1:** Energetics evaluation at T=298.15 K: Interaction energy, $E_{\mathrm{int}}$ and Potential energy PE of RVFV peptide in the solvents.

$x_{1}$:$x_{2}$	$E_{\mathrm{int}}$ (kJ mol${}^{-1}$)	*PE* (kJ mol${}^{-1}$)
100:00	−87,045 ± 1465	−835 ± 155
90:10	−205,431 ± 6910	−1272 ± 179
80:20	−395,309 ± 2395	−1705 ± 185
70:30	−462,843 ± 3344	−2541 ± 176
60:40	−513,576 ± 1553	−2714 ± 273
50:50	−552,467 ± 876	−2252 ± 215
40:60	−589,103 ± 920	−2751 ± 213
30:70	−621,278 ± 900	−3078 ± 246
20:80	−659,920 ± 824	−3702 ± 231
10:90	−694,648 ± 808	−4397 ± 219
6QHG		−8227.469

## Data Availability

Data is contained within the article or supplementary material.

## Supplementary Materials

The following are available online at https://www.mdpi.com/article/10.3390/biom11101427/s1.

## Abbreviations

The following abbreviations are used in this manuscript:

RMSD	Root-Mean-Squared-Deviation
RMSF	Root-Mean-Squared-Fluctuation
MD	Molecular Dynamics
PDB	Protein Data Bank
RVFV	Rift Valley Fever Virus
