# Supplementary material for: Computing the Structural Dynamics of RVFV L Protein Domain in Aqueous Glycerol Solutions
Source: Biomolecules. 2021 Sep 29;11(10):1427. doi: 10.3390/biom11101427 (PMC8533350; doi:10.3390/biom11101427)
Supplement: Supplementary file 1 [file biomolecules-11-01427-s001.zip › biomolecules-1335869-supplementary.pdf]

Supplementary Material for: Computing the Structural Dynamics of RVFV L Protein Domain in Aqueous Glycerol Solutions

Gideon K. Gogovi[1], Swabir Silayi[2], and Amarda Shehu[3,4,5,6]

[1] Department of Mathematics and Statistics, University of Houston - Downtown
[2] Office of Research Computing, George Mason University
[3] Department of Computer Science, George Mason University
[4] Department of Bioengineering, George Mason University
[5] School of Systems Biology, George Mason University
[6] Center for Advancing Human-Machine Partnerships, George Mason University

Summary of Simulations: Glycerol proportion (x_1) and water proportion (x_2).

$x_1 : x_2$	# atoms	Time (ns)	box size/side (Å)
100:00	43849	100×7	71.421
90:10	44230	100×5	71.527
80:20	44611	100×9	71.580
70:30	44992	100×5	71.961
60:40	45373	100×5	72.502
50:50	45754	100×10	73.061
40:60	46135	100×5	73.788
30:70	46516	100×4	74.225
20:80	46897	100×8	74.845
10:90	47278	100×7	75.470

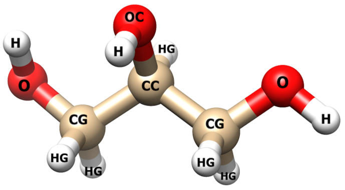

Figure S1: Atom identification in the glycerol molecule.

Density, ρ of glycerol (x_1) : water (x_2) mixtures at 298.15 K and atmospheric pressure.

$x_1 : x_2$	Calculated ρ (g/cm^3)	Experimental ρ (g/cm^3)
100 : 00	1.259 ± 0.002	1.25791
90 : 10	1.253 ± 0.003	1.25331
80 : 20	1.250 ± 0.002	1.24648
70 : 30	1.230 ± 0.001	1.23632
60 : 40	1.202 ± 0.002	1.22565
50 : 50	1.174 ± 0.002	1.21375
40 : 60	1.146 ± 0.001	1.19845
30 : 70	1.119 ± 0.002	1.18300
20 : 80	1.091 ± 0.002	1.14286
10 : 90	1.063 ± 0.002	1.09524

Histogram of Radius of gyration of the domain in glycerol solutions.

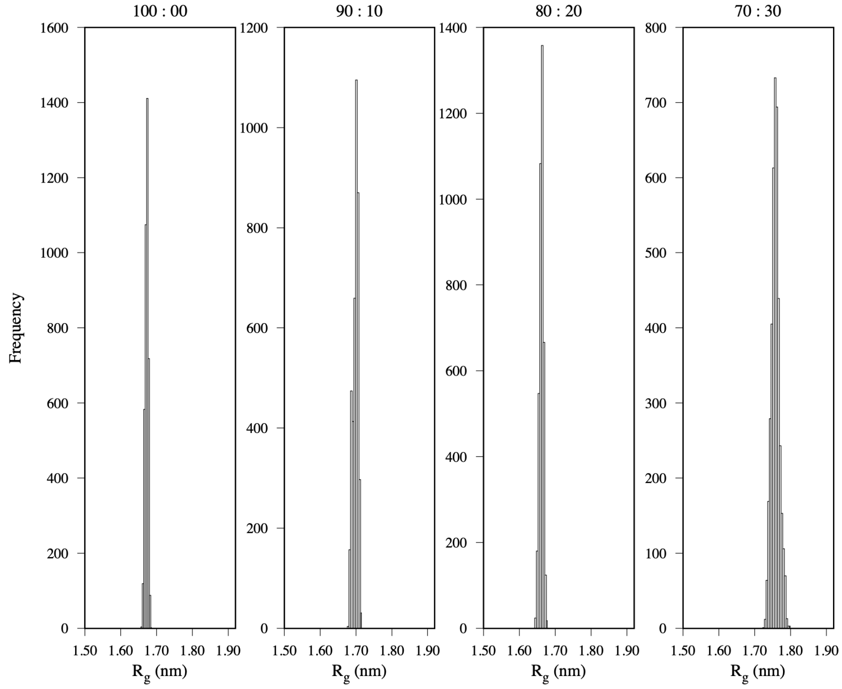

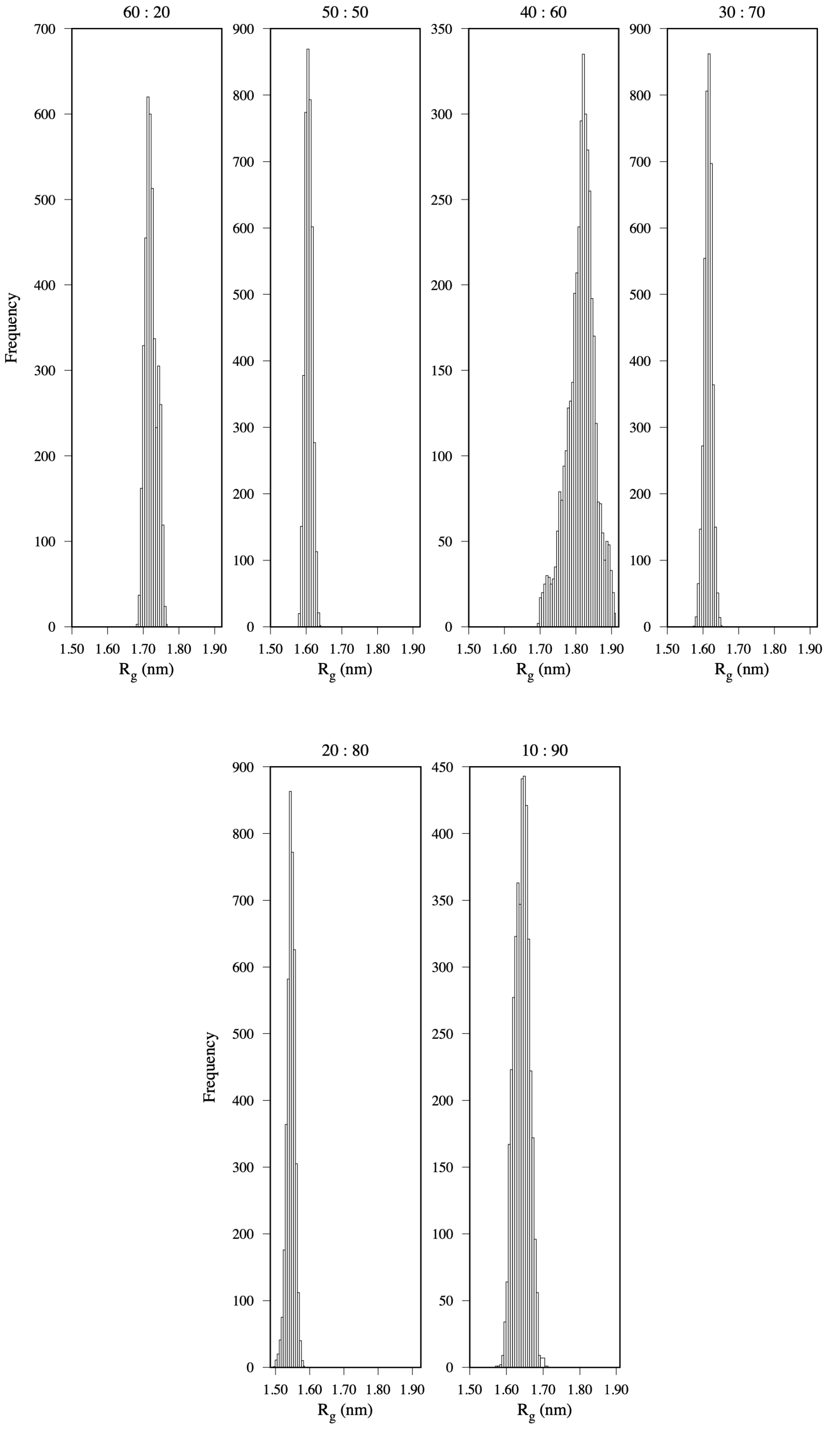
60 : 20
50 : 50
40 : 60
30 : 70
20 : 80
10 : 90
Frequency
R_g (nm)
1.50 1.60 1.70 1.80 1.90

Comparison of cluster sizes of the protein domain: Average distance between clusters within solvents, d_s (nm) and Average distance to centroid, d_c (nm)

$x_1 : x_2$	d_s (nm)	d_c (nm)
100 : 00	0.083 ± 0.002	0.103 ± 0.004
90 : 10	0.096 ± 0.004	0.143 ± 0.008
80 : 20	0.110 ± 0.002	0.157 ± 0.010
70 : 30	0.146 ± 0.066	0.214 ± 0.008
60 : 40	0.018 ± 0.012	0.283 ± 0.011
50 : 50	0.171 ± 0.009	0.262 ± 0.010
40 : 60	0.241 ± 0.020	0.393 ± 0.023
30 : 70	0.184 ± 0.006	0.262 ± 0.016
20 : 80	0.192 ± 0.009	0.264 ± 0.012
10 : 90	0.200 ± 0.026	0.281 ± 0.020

Table S1: Averages of the structural property evaluation of the domain: Root-mean-squared deviation, $RMSD$, Radius of gyration, R^g, Hydrodynamic radius R^{hyd}, End-to-end distance R^{e-e}, and Solvent-accessible surface area, $SASA$.

$x_1 : x_2$	$RMSD$ (nm)	R_g (nm)	R_{hyd} (nm)	R_g/R_{hyd}	R_{e-e} (nm)	$SASA$ (nm^2)
100:00	0.41 ± 0.003	1.67 ± 0.004	3.35 ± 0.01	0.499	2.81 ± 0.03	946.36 ± 30.53
90:00	0.45 ± 0.01	1.70 ± 0.01	3.38 ± 0.01	0.503	2.69 ± 0.05	963.39 ± 6.46
80:00	0.48 ± 0.01	1.66 ± 0.01	3.32 ± 0.01	0.500	2.34 ± 0.21	929.85 ± 36.53
70:00	0.59 ± 0.02	1.76 ± 0.01	3.37 ± 0.01	0.522	3.19 ± 0.15	961.55 ± 27.82
60:00	0.64 ± 0.03	1.72 ± 0.02	3.34 ± 0.01	0.515	2.70 ± 0.30	930.23 ± 56.27
50:00	0.63 ± 0.02	1.61 ± 0.01	3.23 ± 0.01	0.498	3.62 ± 0.31	837.14 ± 40.42
40:00	0.88 ± 0.07	1.81 ± 0.04	3.40 ± 0.02	0.532	3.08 ± 0.54	891.02 ± 76.75
30:00	0.48 ± 0.02	1.61 ± 0.01	3.19 ± 0.02	0.505	2.24 ± 0.09	796.71 ± 32.75
20:00	0.76 ± 0.02	1.54 ± 0.01	3.08 ± 0.01	0.500	2.51 ± 0.10	759.11 ± 66.75
10:00	0.68 ± 0.03	1.64 ± 0.02	3.16 ± 0.02	0.519	1.95 ± 0.37	758.29 ± 39.41
**6QHG**	**0.00**	**1.51**	**2.91**	**0.519**	**2.28**	**673.71**
